# The patient acceptable symptomatic state for commonly used outcome scores 10 years after matrix‐associated autologous chondrocyte implantation

**DOI:** 10.1002/ksa.12661

**Published:** 2025-03-28

**Authors:** Kevin‐Arno Koch, Raphael Trefzer, Mustafa Hariri, Paul Mick, Tilman Walker, Stefanos Tsitlakidis, Johannes Weishorn

**Affiliations:** ^1^ Department of Orthopaedics, Heidelberg University Hospital Ruprecht‐Karls‐University Heidelberg Heidelberg Germany

**Keywords:** cartilage, clinical relevance, knee, patient acceptable symptomatic state, treatment outcome

## Abstract

**Purpose:**

To determine the Patient Acceptable Symptomatic State (PASS) thresholds for the Knee Injury and Osteoarthritis Outcome Score (KOOS), International Knee Documentation Committee (IKDC), Lysholm and EuroQol 5 Dimensions (EQ‐5D) scores and to identify predictors of PASS at 10 years after matrix‐associated autologous chondrocyte implantation (M‐ACI).

**Methods:**

Patients who underwent M‐ACI for chondral defects of the knee between 2011 and 2015 were prospectively evaluated. KOOS, IKDC, Lysholm and EQ‐5D scores and anchor‐based questions were assessed at baseline and at least 10 years post‐operatively. PASS thresholds were determined using receiver operating characteristic (ROC) curve analyses. Multivariable binomial regression analysis was performed to determine the effect of sex, body mass index (BMI), articular cartilage maturation status and the presence of more than one previous knee surgery on the likelihood of achieving PASS.

**Results:**

A total of 112 patients who met the inclusion criteria were evaluated at a mean of 11.3 ± 1.2 years post‐operatively, with a mean age of 29.2 ± 11.0 years. The PASS thresholds for the aforementioned PROMs at 10 years were 61.9 for KOOS (Symptoms 51.8, Pain 79.2, Activities of Daily Living 87.5, Sport 57.5 and Quality of Life 59.4), 59.7 for IKDC, 61.5 for Lysholm and 82.5 for EQ‐5D. The ROC curve showed good to excellent predictive value with an area under the curve (AUC) of 0.76–0.84. Male gender (odds ratio [OR] = 3.1; *p* = 0.016) and BMI between 20 and 29 (OR = 3.9; *p* = 0.004) had a positive predictive value for achieving PASS at long‐term follow‐up.

**Conclusions:**

The present study determined long‐term PASS thresholds for KOOS, IKDC, Lysholm and EQ‐5D scores in patients undergoing M‐ACI for cartilage repair at the knee. Male gender and a BMI of 20–29 were positive predictors of the likelihood of achieving PASS at 10 years. The identified PASS thresholds are critical for assessing clinical outcomes, evaluating procedural efficacy for regulatory considerations, and planning sample sizes for prospective, controlled studies.

**Level of Evidence:**

Level IV.

**Abbreviations**
ADL: activities of daily living
AUC: area under curve
BMI: body mass index
CI: confidence interval
EQ‐5D: EuroQol 5 Dimensions
FU: Follow‐up
ICRS: International Cartilage Research Society
IKDC: International Knee Documentation Committee
IQR: Interquartile Range
KOOS: Knee Injury and OsteoarthritiPs Outcome Score
MCID: Minimal Clinically Important Difference
M‐ACI: matrix‐associated autologous chondrocyte implantation
PASS: patient acceptable symptomatic state
PROMs: Patient‐Reported Outcome Measures
QOL: Quality of Life
ROC: Receiver Operating Characteristic
SD: Standard Deviation

## INTRODUCTION

Patient‐reported outcome measures (PROMs) are widely used to measure the results of surgical interventions and to compare patients' function, pain and quality of life. The Knee Injury and Osteoarthritis Outcome Score (KOOS), the International Knee Documentation Committee (IKDC) score, the Lysholm score and the EuroQol 5 Dimensions (EQ‐5D) score are established instruments for assessing outcomes after cartilage repair of the knee [14, 15, 17, 23]. The minimal clinically important difference (MCID), defined as the smallest difference in score that patients perceive as beneficial, and the patient acceptable symptomatic state (PASS), defined as the level of symptoms above which patients consider themselves well, have been introduced to classify scores and their change over time [19, 33]. They are therefore established parameters in clinical research. While MCID refers to a change in score, PASS refers to an absolute threshold. Both terms are relevant treatment goals and provide critical information to clinicians and researchers, with MCID being of great importance, especially in the perioperative period, and PASS being of significant relevance for the evaluation of both short‐ and long‐term outcomes [4, 31].

Articular cartilage defects are serious pathologies that may be a predisposing factor for the early onset of osteoarthritis [8, 11]. Autologous chondrocyte implantation (ACI) is an established treatment for symptomatic cartilage lesions of the knee to improve patients' pain, function and quality of life and to prevent the onset of osteoarthritis [5, 34]. ACI was introduced in the 1990s and has been continuously developed, with third‐generation matrix‐associated ACI (M‐ACI) currently being widely used in Europe [16, 29]. While for a long time, only short‐ to mid‐term clinical results were available for M‐ACI, several long‐term studies with more than 10 years of follow‐up have recently been published [9, 12, 29]. Although reference values for MCID and PASS are available for the assessment of short‐ and medium‐term clinical outcomes after M‐ACI, long‐term cut‐off values for PASS are lacking [7, 21]. The identification of long‐term PASS threshold values is crucial for the classification of PROMs, the evaluation of the efficacy of the procedure, and the sample size planning of long‐term studies, which are expected to increase in the future. However, little is known about the long‐term PASS threshold values of the KOOS, IKDC, Lysholm and EQ‐5D scores in patients undergoing M‐ACI.

The present study aimed to address this gap by determining the PASS of the aforementioned PROMs and identifying potential predictors of achieving PASS at 10 years in patients undergoing M‐ACI surgery for focal articular cartilage defects of the knee. It was hypothesized that reliable PASS cutoffs and predictors of PASS achievement could be established using an anchor‐based approach.

## MATERIALS AND METHODS

### Study design

The present study uses data from an institutional review board‐approved database of patients who underwent cartilage repair for articular cartilage defects of the knee at a single‐centre academic institution. In this database, all patients are routinely contacted at 1, 2, 5 and every additional 5 years post‐operatively and invited to the single‐centre academic institution for clinical and radiologic review. Written informed consent for study participation and institutional review board approval were obtained before study implementation. This study was conducted in accordance with the 2008 Declaration of Helsinki and was approved by the Ethics Commission of the Medical Centre, University of Heidelberg (S‐029/021).

Inclusion and exclusion criteria were defined prior to study enrolment. The present study included individuals over 16 years of age with a minimum follow‐up of 10 years treated with M‐ACI for symptomatic International Cartilage Regeneration & Joint Preservation Society (ICRS) Grade III or IV articular cartilage defects of the knee [19]. The indication for treatment was symptomatic isolated joint line pain and failure of previous non‐operative management in these patients. Patients with advanced osteoarthritis (Kellgren–Lawrence Grade >2), more than one third meniscal deficit, ligamentous instability, untreated tibiofemoral malalignment of >3°, bipolar cartilage lesions, previous hip or ankle surgery according to the indication criteria for cartilage therapy of the Tissue Regeneration Working Group of the German Society for Orthopaedics and Traumatology (DGOU) at the time of surgery were excluded [19]. In addition, patients who withdrew their informed consent at long‐term follow‐up or were converted to knee replacement surgery were excluded. Thus, 14 patients were excluded, and 3 were lost to follow‐up. Therefore, a consecutive cohort of 112 patients who underwent M‐ACI for unifocal ICRS III or IV articular cartilage defects of the knee was identified between 2011 and 2015.

M‐ACI is a well‐established two‐stage surgical procedure performed as described previously [2]. In all patients, chondrocytes were cultured on a biphasic collagen matrix (Novocart 3D®, TETEC) and implanted via mini‐open arthrotomy 4 weeks after arthroscopic harvest. The collagen matrix was adapted to the cartilage defect and fixed with resorbable sutures and fibrin glue.

### Outcome measures

PROMs were assessed at baseline and at least 10 years post‐operatively using validated questionnaires to determine the KOOS, including subscores, IKDC, Lysholm and EQ‐5D scores [32].

The KOOS was originally developed to assess outcomes in patients with osteoarthritis of the knee but has since been validated and widely used in patients with cartilage surgery [23]. The score is based on a 42‐item questionnaire and ranges from 0 (*worst possible knee symptoms*) to 100 (*no knee symptoms*). The subscores for pain (9 items), symptoms (7 items), activities of daily living (ADL, 17 items), sports and recreation (5 items) and knee‐related quality of life (QOL, 4 items) range from 0 to 100. The IKDC Subjective Knee Evaluation Form consists of 18 items to assess symptoms, daily activities and sports function due to various knee conditions and ranges from 0 to 100 points (*worst* to *best*) [15]. The Lysholm Score is an eight‐item health professional assessment of knee‐specific symptoms such as gait, strain, locking, instability, pain, swelling, stair climbing and squatting, also scored from 0 to 100 [17]. The EQ‐5D is a widely used preference‐based instrument for measuring quality of life and includes five items covering mobility, self‐care, usual activities, pain and depression [14]. It is scaled from 0 to 100.

### Calculation of the PASS

An anchor‐based approach was used to determine the PASS for patients at least 10 years after M‐ACI [7]. All patients were asked at their follow‐up visit whether they considered their current status with respect to ADL, sports activity, pain level, level of functional impairment and quality of life to be satisfactory, which could be answered binomially with yes or no. This anchor question was previously established to determine the PASS for the aforementioned PROMs [28]. A receiver operating characteristic (ROC) analysis was performed to determine optimal cutoff points for the different PROMs. The area under the ROC curve (AUC) was calculated to assess the reliability of the identified cutoff, with an AUC of 0.6–0.7 considered acceptable, 0.7–0.8 considered good and an AUC of 0.8–0.9 considered excellent [10]. Multivariable binomial regression analysis was performed to determine the effect of sex, body mass index (BMI), articular cartilage maturation status, and the presence of more than one previous knee surgery on the likelihood of achieving PASS. A BMI of 20–29, one or fewer previous knee surgeries, and early articular cartilage were previously identified as favourable factors for long‐term clinical outcomes [34, 35]. To further analyze potential predictive factors for achieving PASS at 10 years, the articular cartilage of patients younger than 20 years was considered immature, as previously shown by Schmal et al. [24]. A priori power analysis using a two‐tailed *z* test indicated that 75 patients were required to detect a difference of 0.2 between AUCs with 80% power at the 0.05 significance level. The AUC difference of 0.2 was defined as the difference between the AUC with no discriminatory accuracy (0.5) and the AUC with moderate discriminatory accuracy (0.7). The level of statistical significance was set to *α* = 0.05. Statistics were performed using SPSS software version 27.0 (IBM) and G‐Power 3.1 (Heine University).

## RESULTS

The 112 patients who met the inclusion criteria for the present study were evaluated at a mean of 11.3 ± 1.2 years post‐operatively. The mean age of the patients at the time of surgery was 29.2 ± 11.0 years. The mean BMI of the 58 men and 54 women was 26.5 ± 4.5. The mean defect size was 4.8 ± 2.5 cm², and the defect was primarily located at the medial femoral condyle (45%), followed by the patella (29%), trochlea (18%), lateral femoral condyle (6%) and tibia (2%). In 39 cases (35%), a reduction osteotomy was also performed due to a tibiofemoral malalignment of >3°. This results in a post‐operative tibiofemoral alignment of <3° in all 112 patients. Fifty‐two patients (46%) had previous knee surgery. No patient had concomitant anterior cruciate ligament or collateral ligament reconstruction. The overall baseline and 10‐year scores for the KOOS, KOOS subscales, IKDC score, Lysholm score and EQ‐5D score are shown in Table 1 and improved significantly after M‐ACI. There were no floor effects for any of the outcomes at 10 years, and ceiling effects were low for KOOS (1%), IKDC (0%), Lysholm (1%) and EQ‐5D (4%).

**Table 1:** PROMs at baseline and 10 years.

	Baseline			10 years		
Score	Mean ± SD	Median (IQR)	Range	Mean ± SD	Median (IQR)	Range
KOOS	47.1 ± 26.1	42.0 (26.0–61.3)	9.1–100	70.1 ± 16.6	71.7 (59.1–82.7)	27.0–100
Symptoms	23.2 ± 18.4	17.9 (8.9–28.6)	5.8–93.8	56.2 ± 15.2	57.1 (46.4–67.0)	17.9–100
Pain	31.9 ± 22.7	26.7 (14.3–43.2)	7.6–100	80.8 ± 18.5	86.1 (67.4–97.2)	22.2–100
ADL	65.9 ± 35.3	82.4 (28.7–93.4)	10.3–100	87.7 ± 14.5	92.6 (83.8–98.5)	35.3–100
Sport	38.2 ± 25.1	33.8 (20.0–55.0)	0–100	63.0 ± 28.7	65.0 (41.3–90.0)	0–100
QOL	36.2 ± 24.1	31.3 (16.4–54.7)	0–100	63.0 ± 20.7	62.5 (50.0–68.8)	0–100
IKDC	46.1 ± 32.9	44.8 (12.1–78.8)	0–100	72.3 ± 16.7	75.9 (60.9–85.1)	31.0–95.4
Lysholm	47.3 ± 26.0	50.7 (22.2–63.6)	5.8–100	73.0 ± 20.3	75.0 (58.3–90.0)	21.0–100
EQ‐5D	53.0 ± 27.5	59.5 (26.7–72.0)	1.6–100	81.7 ± 17.0	87.5 (75.0–95.0)	2.0–100

Abbreviations: ADL, activities of daily living; EQ‐5D, EuroQol 5 Dimensions; IKDC, International Knee Documentation Committee; IQR, interquartile range; KOOS, Knee Injury and Osteoarthritis Outcome Score; PROM, patient‐reported outcome measure; QOL, quality of life; SD, standard deviation.

### PASS thresholds

The ROC analysis yielded PASS thresholds for the previously mentioned PROMs at 10‐year follow‐up, as shown in Table 2.

**Table 2:** Identifying PASS thresholds of the KOOS, IKDC score, Lysholm score and EQ‐5D score.

	Mean ± SD		ROC analysis	
Score	Failed PASS	Achieved PASS	Optimal cutoff score	AUC
KOOS	49.8 ± 9.9	78.6 ± 10.2	61.9	0.81
Symptoms	45.1 ± 14.7	60.8 ± 12.9	51.8	0.64
Pain	58.3 ± 14.0	90.2 ± 10.1	79.2	0.82
ADL	70.9 ± 15.3	94.7 ± 6.0	87.5	0.77
Sport	28.9 ± 17.8	77.2 ± 18.7	57.5	0.82
QOL	45.8 ± 15.6	70.2 ± 18.2	59.4	0.83
IKDC	52.1 ± 10.5	80.8 ± 10.3	59.7	0.80
Lysholm	49.6 ± 14.6	82.8 ± 13.0	61.5	0.76
EQ‐5D	68.5 ± 16.6	87.2 ± 13.8	82.5	0.84

Abbreviations: ADL, activities of daily living; AUC, area under curve; EQ‐5D, EuroQol 5 Dimensions; IKDC, International Knee Documentation Committee; KOOS, Knee Injury and Osteoarthritis Outcome Score; PASS, patient acceptable symptomatic state; QOL, quality of life; ROC, receiver operating characteristic; SD, standard deviation.

**Figure 1:**
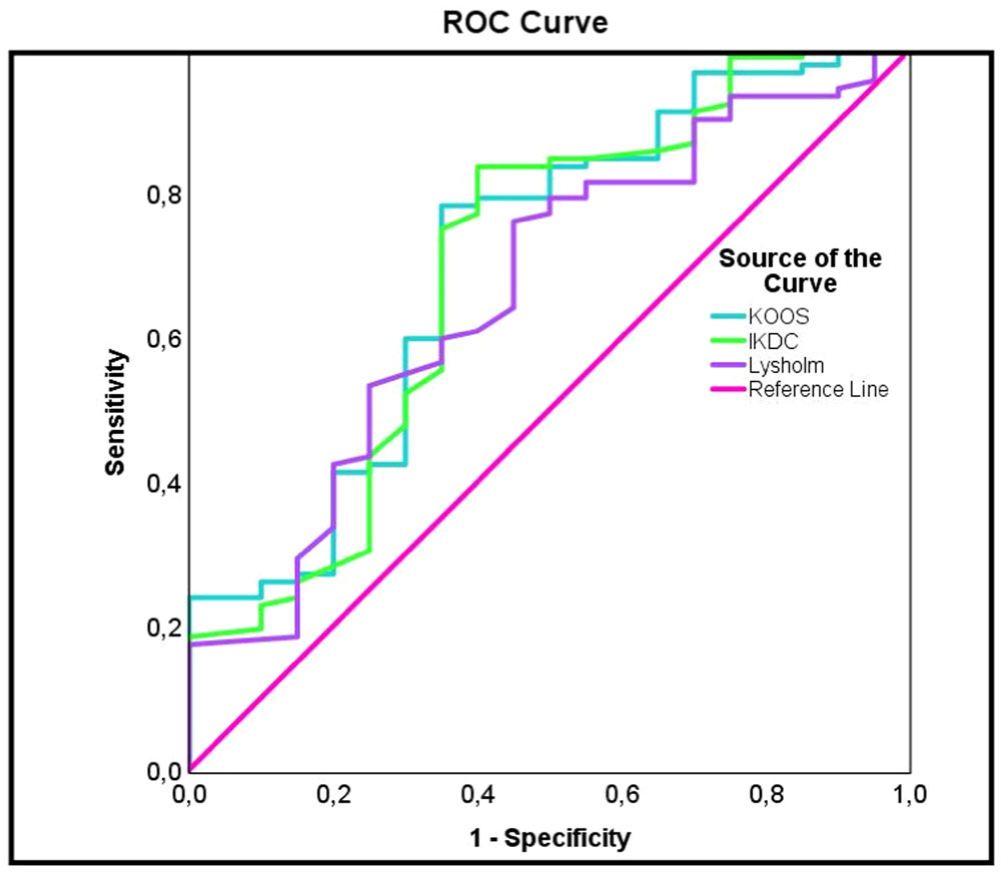
ROC curve determining PASS and its reliability on functional PROMs at 10 years after M‐ACI. IKDC, International Knee Documentation Committee; KOOS, Knee Injury and Osteoarthritis Outcome Score; M‐ACI, matrix‐associated autologous chondrocyte implantation; PASS, patient acceptable symptomatic state; PROM, patient‐reported outcome measure; ROC, receiver operating characteristic.

**Figure 2:**
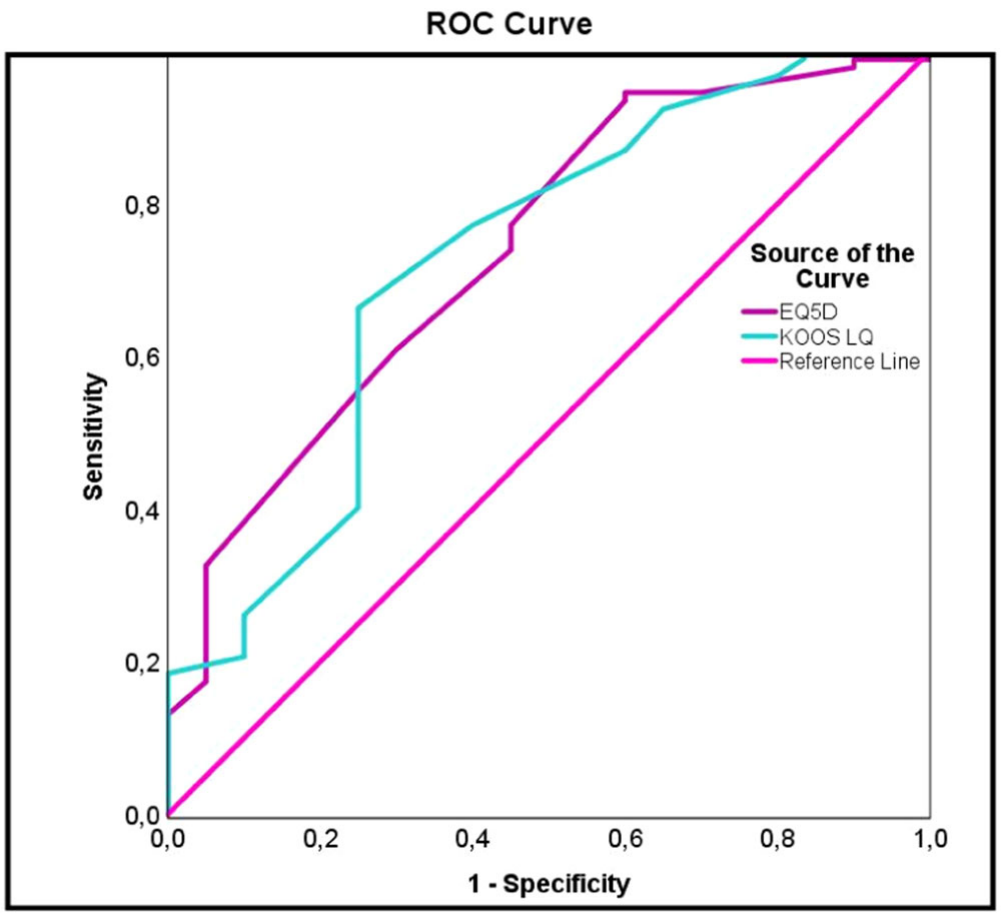
ROC curve determining PASS and its reliability on quality of life at 10 years after M‐ACI. EQ‐5D, EuroQol 5 Dimensions; KOOS, Knee Injury and Osteoarthritis Outcome Score; M‐ACI, matrix‐associated autologous chondrocyte implantation; PASS, patient acceptable symptomatic state; ROC, receiver operating characteristic.

The ROC curve showed an acceptable to excellent predictive value of the newly identified PASS threshold with AUCs above 0.7 for functional scores (Figure 1) and quality of life scores (Figure 2).

When stratified by gender, women tend to show slightly lower PASS cut‐off scores for KOOS, IKDC and Lysholm (Table 3). On the EQ‐5D, however, women appear to require a higher score to achieve a PASS. The AUCs for the PROMs were higher for women than for men, although the reliability of the cut‐off scores was acceptable for both sexes.

**Table 3:** Identification of gender differences of PASS thresholds of the KOOS, IKDC score, Lysholm score and EQ‐5D score.

	Male		Female	
Score	Optimal cutoff score	AUC	Optimal cutoff score	AUC
KOOS	61.8	0.73	60.5	0.90
Symptoms	51.8	0.63	55.4	0.62
Pain	70.8	0.79	79.1	0.87
ADL	88.9	0.68	86.7	0.90
Sport	57.5	0.75	52.5	0.91
QOL	59.4	0.74	53.1	0.83
IKDC	64.9	0.69	59.7	0.90
Lysholm	62.0	0.68	58.5	0.85
EQ‐5D	75.5	0.72	82.5	0.88

Abbreviations: ADL, activities of daily living; AUC, area under curve; EQ‐5D, EuroQol 5 Dimensions; IKDC, International Knee Documentation Committee; KOOS, Knee Injury and Osteoarthritis Outcome Score; PASS, patient acceptable symptomatic state; QOL, quality of life.

Overall, across all scores and subscales, more than two thirds of patients achieved PASS at long‐term follow‐up. A majority of patients achieved PASS on the IKDC, KOOS and Lysholm scores, with 76%, 71% and 71%, respectively. Additionally, 62% of patients attained an acceptable quality of life score on the EQ‐5D score.

### Influence of demographic characteristics on PASS achievement

Multivariate analysis showed that the male sex (odds ratio [OR] = 3.1; *p* = 0.016) and a BMI between 20 and 29 (OR = 3.9; *p* = 0.004) had a positive predictive value for achieving PASS at long‐term follow‐up (Table 4). Immature articular cartilage in adolescents and one or fewer previous knee surgeries showed a positive trend for the likelihood of achieving PASS. However, the binary‐logistic association was not significant (*p* > 0.05).

**Table 4:** Influence of patient‐specific characteristics on the likelihood of achieving PASS (*R*² = 0.23).

Patient‐specific characteristics	Odds ratio (95% CI)	*p*
Male sex	3.1 (1.2–7.7)	0.016*
Immature cartilage	1.9 (0.7–5.7)	0.229
BMI of 20–29	3.9 (1.5–9.9)	0.004*
More than 1 previous knee surgery	0.5 (0.1–1.6)	0.246

Abbreviations: BMI, body mass index; CI, confidence interval; PASS, patient acceptable symptomatic state.

* Indicates significance.

## DISCUSSION

The main finding of the present study was the identification of clinically relevant thresholds for PASS at 10 years after M‐ACI for KOOS, IKDC score, Lysholm score and EQ‐5D score, reflecting four widely used PROMs in cartilage repair. In addition, men and patients with a BMI between 20 and 29 were more likely to achieve PASS at long‐term follow‐up. These findings are critical to the scientific debate as they provide important benchmarks for upcoming clinical trials investigating long‐term performance after M‐ACI surgery. They allow for individualized consideration of clinical outcomes without group comparisons and provide the odds of achieving long‐term PASS, allowing for improved evidence‐based patient education.

M‐ACI is a widely used procedure for cartilage repair of articular cartilage defects in the knee. Although there are a few studies reporting clinically relevant changes in various PROMs up to 2 years, evidence on PASS is scarce [30]. In particular, long‐term follow‐up data are lacking. Ogura et al. reported the MCID and substantial clinical benefit (SCB), defined as the value perceived by patients as substantial clinical improvement, for KOOS, IKDC score, Lysholm and SF‐12 at 2 years after M‐ACI in 92 patients [21]. The MCID for KOOS was 10 with variation among subscores, 10.8–16.4 for IKDC, 4.2–10.5 for Lysholm, 6.2–8.2 for SF‐12 physical and 4.2–10.5 for SF‐12 mental component summary [21]. The PASS was not calculated, and statistical analysis varied between anchor and distribution‐based approaches between different PROMs. Another analysis by Chahal et al. reported clinically important differences (CID) and PASS scores in a cohort of 113 patients 1 year after various cartilage repair procedures [7]. The CID is defined as a change that holds clinical significance based on the method of measurement but is not necessarily minimal [20]. CID scores for KOOS subscores ranged from 8.3 for pain to 30 for sport, while PASS scores ranged from 43.8 for sport to 86.8 for ADL. Other PASS scores ranged from 62.1 for the IKDC score to 70.0 for the Lysholm score using an anchor‐based approach [7]. The anchor‐based method and the large sample size (*n* = 113) were strengths of the study by Chahal et al. [7]. However, the internal validity was limited by the inclusion of patients with different cartilage repair techniques. In contrast, the high internal validity due to the focus on M‐ACI was one of the strengths of the present study. The present study found comparable PASS scores ranging from 52.5 for KOOS Sport, 86.7 for KOOS ADL, 59.7 for IKDC and 58.5 for Lysholm scores. This was an interesting finding because the PASS of patients after M‐ACI did not decrease in the long term. In contrast, several studies evaluating PASS in patients after unicondylar (UKA) or total knee arthroplasty (TKA) have shown that PASS thresholds decrease over the long term [22, 27]. The younger patient population in M‐ACI, which remains highly demanding in terms of symptoms, function and quality of life, even at long‐term follow‐up, may explain this difference. Another aspect of the present study was that 76%, 71% and 71% of patients achieved PASS on the IKDC, KOOS and Lysholm scores, respectively. In the literature, PASS on the IKDC score in the study by Chahal et al. was 50%, while Ogura reported an SCB of 47% [7]. The remarkable difference to the present study may be due to the heterogeneity of the surgical procedures or the variation of the endpoints.

The fact that women tend to have lower PASS thresholds on clinical scores such as KOOS, IKDC and Lysholm was another interesting finding of the present study. However, the PASS threshold on the EQ‐5D, which reflects a quality of life score, was higher in women. This is consistent with the literature, where men have comparable to slightly higher functional scores than women, while women tend to have greater improvements in PROMs [13]. Women undergoing orthopaedic surgery report more pain and lower functional scores than men preoperatively, but these differences often diminish post‐operatively [25]. Reasons include biological and psychosocial factors as well as health status and comorbidities. In a large registry‐based analysis focusing on gender differences in PROMs after cartilage surgery, women were less satisfied, had higher re‐intervention rates, and reported lower quality of life compared to men [18]. This may be explained by the fact that women tend to be more sensitive to their own health on self‐reported measures [1]. In the World Health Survey, women reported significantly worse health than men on all self‐reported health indicators [3]. In addition, there are many confounding factors in self‐reported knee functional scores, such as preoperative mental health, for which gender bias in self‐reporting has been demonstrated [26]. In particular, men underreport depressive symptoms. Nevertheless, the differences in individual PASS thresholds in men and women identified in the present study may help to evaluate clinical outcomes after M‐ACI in a gender‐specific perspective in the future, thus allowing for a more differentiated analysis of clinical outcomes after M‐ACI. In addition to gender, where men had 3.1‐fold increased odds of achieving PASS, a BMI between 20 and 29, which has recently been identified as a factor influencing clinical outcome in M‐ACI, was associated with 3.9‐fold increased odds of achieving PASS [6, 35].

The present study contributes to the existing literature by being the first of its kind to report on long‐term PASS thresholds for functional scores 10 years after M‐ACI, which are critical in evaluating long‐term clinical trials. Patient characteristics, particularly gender and BMI, were confirmed to be important predictors of PASS thresholds at 10 years. In addition, the PASS cutoffs identified in this study showed good to excellent reliability, with AUC estimates ranging from 0.76 to 0.84.

Limitations of the current study include the limited external validity of the PASS threshold. Focusing on patients following M‐ACI surgery allows for high comparability with future studies investigating clinical outcomes after M‐ACI. However, generalizability to other cartilage repair procedures was limited. In addition, the present study included data from patients who had previously undergone knee surgery or concomitant osteotomy. Although this reflects the reality of care in daily practice to provide optimal biomechanical loading for graft maturation, it may confound the results of the study. Another limitation was the lack of reporting MCID values, which was explained by their limited significance for long‐term clinical outcomes and the already published reference values up to 2 years post‐operatively. Furthermore, although the anchor‐based method has many advantages over the distribution‐based method, it still has some limitations, especially recall bias. In addition, we did not consider the effect of several factors, especially mental health status, that may bias PROMs and thus PASS thresholds. However, the baseline characteristics of the collective studies showed comparable characteristics to outcome studies on cartilage repair.

## CONCLUSION

The present study was the first to show long‐term PASS thresholds for KOOS, IKDC, Lysholm and EQ‐5D scores in patients undergoing M‐ACI for cartilage repair at the knee. Male gender and a BMI of 20–29 were positive predictors of the likelihood of achieving PASS at 10 years. The identified PASS thresholds are critical for assessing clinical outcomes, evaluating procedure efficacy for regulatory considerations, and sample size planning for prospective, controlled studies.

## AUTHOR CONTRIBUTIONS

Kevin‐Arno Koch performed data extraction and statistical analysis and drafted the manuscript. Raphael Trefzer, Mustafa Hariri, Paul Mick, Tilman Walker and Stefanos Tsitlakidis critically revised the manuscript. Johannes Weishorn supervised the study, performed data extraction and statistical analysis, and drafted the manuscript.

## CONFLICT OF INTEREST STATEMENT

The authors declare no conflicts of interest.

## ETHICS STATEMENT

The current study was approved by the Ethics Commission of the Medical Center, University of Heidelberg (S‐029/021). Written informed consent was obtained from every patient before inclusion.

## Data Availability

The data will be available upon reasonable request.

## References

[1] Abed V , Kapp S , Nichols M , Shephard L , Jacobs C , Conley C , et al. Responsiveness of patient‐reported outcome measures after large knee articular cartilage transplantation: a systematic review and meta‐analysis. Am J Sports Med. 2024;52:2676–2682. 38264794 10.1177/03635465231196156

[2] Barié A , Kruck P , Sorbi R , Rehnitz C , Oberle D , Walker T , et al. Prospective long‐term follow‐up of autologous chondrocyte implantation with periosteum versus matrix‐associated autologous chondrocyte implantation: a randomized clinical trial. Am J Sports Med. 2020;48:2230–2241. 32667270 10.1177/0363546520928337

[3] Boerma T , Hosseinpoor AR , Verdes E , Chatterji S . A global assessment of the gender gap in self‐reported health with survey data from 59 countries. BMC Public Health. 2016;16:675. 27475755 10.1186/s12889-016-3352-y PMC4967305

[4] Boffa A , Andriolo L , Franceschini M , Di Martino A , Asunis E , Grassi A , et al. Minimal clinically important difference and patient acceptable symptom state in patients with knee osteoarthritis treated with PRP injection. Orthop J Sports Med. 2021;9:23259671211026242. 10.1177/23259671211026242 34631901 PMC8495529

[5] Bumberger A , Niemeyer P , Angele P , Wright EK , Faber SO . Hydrogel‐based and spheroid‐based autologous chondrocyte implantation of the knee show similar 2‐year functional outcomes: an analysis based on the German Cartilage Registry (KnorpelRegister DGOU). Knee Surg Sports Traumatol Arthrosc. 2024;32:2258–2266. 38751089 10.1002/ksa.12248

[6] Bumberger A , Rupp MC , Lattermann C , Kleiner A , Niemeyer P . Increased risk of reoperation and failure to attain clinically relevant improvement following autologous chondrocyte implantation of the knee in female patients and individuals with previous surgeries: a time‐to‐event analysis based on the German cartilage registry (KnorpelRegister DGOU). Knee Surg Sports Traumatol Arthrosc. 2023;31:5837–5847. 37950850 10.1007/s00167-023-07615-5 PMC10719132

[7] Chahal J , Lansdown DA , Davey A , Davis AM , Cole BJ . The clinically important difference and patient acceptable symptomatic state for commonly used patient‐reported outcomes after knee cartilage repair. Am J Sports Med. 2020;49:193–199. 33226845 10.1177/0363546520969883

[8] Colombini A , Libonati F , Lopa S , Peretti GM , Moretti M , de Girolamo L . Autologous chondrocyte implantation provides good long‐term clinical results in the treatment of knee osteoarthritis: a systematic review. Knee Surg Sports Traumatol Arthrosc. 2023;31:2338–2348. 35716187 10.1007/s00167-022-07030-2

[9] Colombini A , Raffo V , Gianola S , Castellini G , Filardo G , Lopa S , et al. Matrix‐assisted autologous chondrocyte transplantation is effective at mid/long‐term for knee lesions: a systematic review and meta‐analysis. Knee Surg Sports Traumatol Arthrosc. 2024. 10.1002/ksa.12549 PMC12310092 39624924

[10] Dekhne MS , Fontana MA , Pandey S , Driscoll DA , Lyman S , McLawhorn AS , et al. Defining patient‐relevant thresholds and change scores for the HOOS JR and KOOS JR anchored on the patient‐acceptable symptom state question. Clin Orthop Relat Res. 2024;482:688–698. 37773026 10.1097/CORR.0000000000002857 PMC10936968

[11] Dhillon J , Decilveo AP , Kraeutler MJ , Belk JW , McCulloch PC , Scillia AJ . Third‐generation autologous chondrocyte implantation (cells cultured within collagen membrane) is superior to microfracture for focal chondral defects of the knee joint: systematic review and meta‐analysis. Arthroscopy. 2022;38:2579–2586. 35283221 10.1016/j.arthro.2022.02.011

[12] Ebert JR , Zheng M , Fallon M , Wood DJ , Janes GC . 10‐year prospective clinical and radiological evaluation after matrix‐induced autologous chondrocyte implantation and comparison of tibiofemoral and patellofemoral graft outcomes. Am J Sports Med. 2024;52:977–986. 38384192 10.1177/03635465241227969 PMC10943616

[13] Faber S , Zinser W , Angele P , Spahn G , Löer I , Zellner J , et al. Does gender influence outcome in cartilage repair surgery? An analysis of 4,968 consecutive patients from the German Cartilage Registry (Knorpel Register DGOU). Cartilage. 2021;13:837S–845S. 32476447 10.1177/1947603520923137 PMC8808879

[14] Hurst NP , Kind P , Ruta D , Hunter M , Stubbings A . Measuring health‐related quality of life in rheumatoid arthritis: validity, responsiveness and reliability of EuroQol (EQ‐5D). Rheumatology. 1997;36:551–559. 10.1093/rheumatology/36.5.551 9189057

[15] Irrgang JJ , Anderson AF , Boland AL , Harner CD , Kurosaka M , Neyret P , et al. Development and validation of the International Knee Documentation Committee subjective knee form. Am J Sports Med. 2001;29:600–613. 11573919 10.1177/03635465010290051301

[16] Kunze KN , Persaud S , Briano J , Rodeo SA , Warren RF , Wickiewicz TL , et al. Outcomes of revision cartilage restoration surgery for failed primary treatment of chondral or osteochondral defects of the knee: a systematic review. Am J Sports Med. 2025. 10.1177/03635465241260271 39757922

[17] Lysholm J , Gillquist J . Evaluation of knee ligament surgery results with special emphasis on use of a scoring scale. Am J Sports Med. 1982;10:150–154. 6896798 10.1177/036354658201000306

[18] Mai C , Mai P , Hinz M , Saenger R , Seil R , Tischer T , et al. Females show worse functional outcome and quality of life compared to males 2 years after meniscus surgery: data analysis from the German Arthroscopy Registry. Knee Surg Sports Traumatol Arthrosc. 2024;32:2644–2654. 38454792 10.1002/ksa.12131

[19] Niemeyer P , Albrecht D , Aurich M , Becher C , Behrens P , Bichmann P , et al. Empfehlungen der AG Klinische Geweberegeneration zur Behandlung von Knorpelschäden am Kniegelenk. Z Orthop Unfall. 2023;161:57–64. 35189656 10.1055/a-1663-6807

[20] Norman GR , Sloan JA , Wyrwich KW . Interpretation of changes in health-related quality of life: the remarkable universality of half a standard deviation. Med Care. 2003;41(5):582–592. 10.1097/01.MLR.0000062554.74615.4C 12719681

[21] Ogura T , Ackermann J , Barbieri Mestriner A , Merkely G , Gomoll AH . Minimal clinically important differences and substantial clinical benefit in patient‐reported outcome measures after autologous chondrocyte implantation. Cartilage. 2020;11:412–422. 30221977 10.1177/1947603518799839 PMC7488950

[22] Plancher KD , Brite JE , Briggs KK , Petterson SC . Patient‐acceptable symptom state for reporting outcomes following unicompartmental knee arthroplasty: a matched pair analysis comparing UKA in ACL‐deficient versus ACL‐intact knees. Bone Joint J. 2021;103–b:1367–1372. 10.1302/0301-620X.103B8.BJJ-2021-0170.R1 34334042

[23] Roos EM , Lohmander LS . The Knee injury and Osteoarthritis Outcome Score (KOOS): from joint injury to osteoarthritis. Health Qual Life Outcomes. 2003;1:64. 14613558 10.1186/1477-7525-1-64 PMC280702

[24] Schmal H , Pestka JM , Salzmann G , Strohm PC , Südkamp NP , Niemeyer P . Autologous chondrocyte implantation in children and adolescents. Knee Surg Sports Traumatol Arthrosc. 2013;21:671–677. 22552618 10.1007/s00167-012-2036-0

[25] Segal NA , Nilges JM , Oo WM . Sex differences in osteoarthritis prevalence, pain perception, physical function and therapeutics. Osteoarthr Cartil. 2024;32:1045–1053. 10.1016/j.joca.2024.04.002 38588890

[26] Stone AV , Murphy ML , Jacobs CA , Lattermann C , Hawk GS , Thompson KL , et al. Mood disorders are associated with increased perioperative opioid usage and health care costs in patients undergoing knee cartilage restoration procedure. Cartilage. 2022;13:19476035221087703. 35333656 10.1177/19476035221087703 PMC9137305

[27] Tan YCJ , Chen JYQ , Tay DKJ , Lo NN , Yeo S , Liow MHL . Patient acceptable symptom state thresholds for the Knee Society Score, Oxford Knee Score, and 36‐item short form survey ten years following unicompartmental knee arthroplasty. J Arthroplasty. 2024;39:1480–1486. 38081552 10.1016/j.arth.2023.12.013

[28] Tubach F , Ravaud P , Baron G , Falissard B , Logeart I , Bellamy N , et al. Evaluation of clinically relevant states in patient reported outcomes in knee and hip osteoarthritis: the patient acceptable symptom state. Ann Rheum Dis. 2005;64:34–37. 15130902 10.1136/ard.2004.023028 PMC1755171

[29] Wang AS , Nagelli CV , Lamba A , Saris DBF , Krych AJ , Hevesi M . Minimum 10‐year outcomes of matrix‐induced autologous chondrocyte implantation in the knee: a systematic review. Am J Sports Med. 2024;52(9):2407–2414. 10.1177/03635465231205309 38312085 PMC12456864

[30] Wang D , Chang B , Coxe FR , Pais MD , Wickiewicz TL , Warren RF , et al. Clinically meaningful improvement after treatment of cartilage defects of the knee with osteochondral grafts. Am J Sports Med. 2019;47:71–81. 30481044 10.1177/0363546518808030

[31] Weishorn J , Koch KA , Zietzschmann S , Trefzer R , Walker T , Renkawitz T , et al. Neutral to slightly undercorrected mechanical leg alignment provides superior long‐term results in patients undergoing matrix‐associated autologous chondrocyte implantation. Knee Surg Sports Traumatol Arthrosc. 2024;32:2040–2051. 38738859 10.1002/ksa.12226

[32] Weishorn J , Niemeyer P , Angele P , Spahn G , Tischer T , Renkawitz T , et al. Secondary matrix‐associated autologous chondrocyte implantation after failed cartilage repair shows superior results when combined with autologous bone grafting: findings from the German Cartilage Registry (KnorpelRegister DGOU). Knee Surg Sports Traumatol Arthrosc. 2024. 10.1002/ksa.12467 PMC12022828 39279220

[33] Weishorn J , Tischer T , Niemeyer P , Renkawitz T , Bangert Y . The role of autologous bone grafting in matrix‐associated autologous chondrocyte implantation at the knee: results from the German Cartilage Registry (KnorpelRegister DGOU). Knee Surg Sports Traumatol Arthrosc. 2024;32:929–940. 38426599 10.1002/ksa.12106

[34] Weishorn J , Wiegand J , Koch KA , Trefzer R , Renkawitz T , Walker T , et al. Favourable clinical outcomes and low revision rate after M‐ACI in adolescents with immature cartilage compared to adult controls: results at 10 years. Knee Surg Sports Traumatol Arthrosc. 2025;33:167–176. 39010715 10.1002/ksa.12359 PMC11716355

[35] Weishorn J , Wiegand J , Zietzschmann S , Koch KA , Rehnitz C , Renkawitz T , et al. Factors influencing long‐term outcomes after matrix‐induced autologous chondrocyte implantation: long‐term results at 10 years. Am J Sports Med. 2024;52:2782–2791. 39276119 10.1177/03635465241270152 PMC11409559

